# Supplementary material for: Inhibitory Properties of Aldehydes and Related Compounds against Phytophthora infestans—Identification of a New Lead
Source: Pathogens. 2020 Jul 7;9(7):542. doi: 10.3390/pathogens9070542 (PMC7400633; doi:10.3390/pathogens9070542)

Supplementary Information

Inhibitory properties of aldehydes and related compounds against *Phytophthora infestans* – identification of a new lead.

1. *Synthesis of Ellipticine framework* *S-2*
2. *NMR data* *S-5*
3. *Mycelial Growth Inhibition of cinnamaldehyde (**7**), hydrocinnamaldehyde **6** and Mancozeb **1*** *S-16*

Synthesis of Ellipticine

1-(Phenylsulfonyl)-1*H*-indole

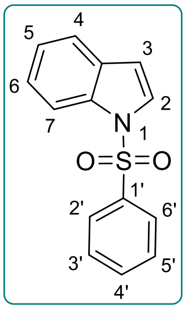

To a solution of indole (25.5 g, 214.0 mmol) and tetrabutylammonium hydrogen sulfate (2.20 g, 6.50 mmol) in toluene (250 mL) was added aqueous sodium hydroxide (50% w/v, 250 mL) at 0 °C to form a biphasic mixture. Benzenesulphonyl chloride (33.2 mL, 260 mmol) was then added and the reaction stirred for 16 hours at room temperature. The aqueous layer was separated and extracted with toluene (2 × 150 mL). Combined organic layers were washed with 1 M hydrochloric acid (2 × 150 mL) and brine (2 × 150 mL), dried over magnesium sulfate and evaporated under reduced pressure. Recrystallisation from dichloromethane and hexane yielded pink crystals (51.43 g, 78.6%); m.p. 77-78 °C (Lit. 77-79 °C)[1]; IR ν_{max}/cm^{-1} : 3139, 3116, 1446, 1370, 1336, 1168, 1120, 709; δ_H (300 MHz, $CDCl_3$): 6.66 (1H, dd, *J* 3.7, 0.8, H-3), 7.22 (1H, overlapping ddd, *J* 8.2, 7.3, 1.1, H-6), 7.31 (1H, overlapping ddd, *J* 8.2, 7.3, 1.1, H-5), 7.39-7.45 (2H, m, H-3′, H-5′), 7.48-7.55 (2H, m, H-7, H-4′), 7.56 (1H, d, *J* 3.7, H-2), 7.85-7.90 (2H, m, H-2′, H-6′), 7.99 (1H, dd, *J* 8.2, 1.1, H-4); m/z (ESI^+): 258 [$(M+H)^+$, 50%]

Pyridine-3,4-dicarboxylic acid anhydride

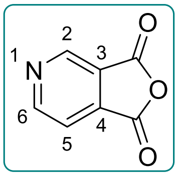

A suspension of pyridine-3-4-dicarboylic acid (25.0 g, 149.60 mmol) in acetic anhydride (85 mL) was heated under nitrogen at 110 °C until the solid dissolved. The solution was then heated to reflux for 40 minutes. Upon cooling to room temperature, the solvent was removed under reduced pressure resulting in a dark brown residue. Ice cold diethyl ether (20 mL) was added to the product and stirred before isolation by vacuum filtration. The product was further washed with diethyl ether (2 × 10 mL) to result in an off-white solid and stored under high-vac until needed (20.0 g, 88.0 %); m.p. 76-78 °C (Lit. 76-77 °C)[1]; IR ν_{max}/cm^{-1}: 1864, 1787, 1283, 1213, 894; δ_H (300 MHz, $CDCl_3$): 7.93 (1H, dd, *J* 4.9, 1.2, H-6), 9.24 (1H, d, *J* 4.9, H-5), 9.38 (1H,d, *J* 1.2, H-2).

4-[{(1-(Phenylsulfonyl)-1*H*-indol-2-yl}carbonyl]-3-pyridinecarboxylic acid and 3-[{(1-(Phenylsulfonyl)-1*H*-indol-2-yl}carbonyl)-3-pyridinecarboxylic acid

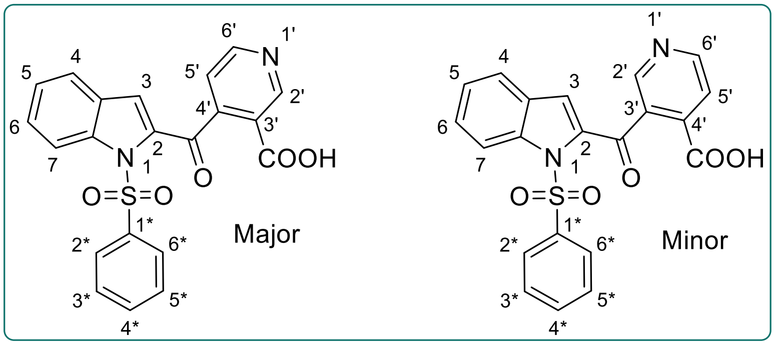

Note: This reaction was carried out in a 1 L 3-necked round bottom flask, equipped with an addition funnel and overhead stirrer.

A solution of diisopropylamine (16.9 mL, 120.0 mmol) in anhydrous THF (150 mL) at -78 °C, under nitrogen was treated with *n*-BuLi (44.0 mL, 2.5 M, 110.0 mmol) *via* cannula. This was maintained at -78 °C for 30 minutes, generating lithium diisopropylamide. 1-(Phenylsulfonyl)-1*H*-indole (25.7g, 100.0 mmol) dissolved in anhydrous THF (80 mL) was added from the addition funnel over 15 minutes, maintaining the temperature below -78 °C. The reaction mixture was let gradually warm to 15 °C over 3 hours, resulting in a white precipitate observed in an orange solution. The reaction mixture was then cooled to -105 °C, the reaction mixture was treated with a solution of pyridine-3,4-dicarboxylic acid anhydride (17.85 g, 120.0 mmol) in anhydrous THF (190 mL) *via* the addition funnel. The solution was added as quickly as possible while maintaining the temperature below -105 °C. The reaction mixture was stirred and allowed to warm to room temperature overnight. The reaction was quenched with water (15 mL) and the solvent was removed under reduced pressure resulting in a dark brown residue. Water (300 mL) was added to the residue resulting in a

fine suspension which was acidified to pH 2 with 37% aqueous hydrochloric acid. The resulting precipitate was isolated by vacuum filtration and washed with water (200 mL) and dried at 50 °C for 24 hours to give the mixture of isomers as a cream solid. The crude product was purified by boiling in acetic acid (50 mL) for 1 hour and filtered once cooled. The filtrate was treated with water until it remained cloudy forming a second crop of product. The 2 crops were combined and multiple fractional recrystallisations from acetone were performed to isolate each isomer, as the minor isomer is less soluble. Combined major isomer yielded 23.3 g (63.1%) and minor isomer yielded 6.4 g (17.3%), giving a total yield of 29.7 g (80.4%)

Major isomer: 4-(1-(Phenylsulfonyl)-1*H*-indole-2-carbonyl)nicotinic acid: (23.3 g, 63.1%) m.p. 259- 260 °C (Lit. m.p. 263-264 °C)[1]; IR ν_{max}/cm^{-1} : 3053, 1677, 1362, 1339, 1310, 1264, 1208, 1090, 1050, 723; δ_H (300 MHz, DMSO-*d*$_6$): 7.26 (1H, s, H-3), 7.38 (1H, ddd, *J* 7.9, 7.3, 0.7 H-6), 7.53-7.64 (2H, m, H-5, H-5′), 7.64-7.83 (4H, m, H-7, H-3*, H-4*, H-5*), 8.13-8.24 (3H, m, H-4, H-2*, H-6*), 8.93 (1H, d, *J* 5.0, H-6′), 9.12 (1H, s, H-2′); m/z (ESI$^+$) 407 [(M+H)$^+$, 100%]

Minor isomer: 3-(1-(Phenylsulfonyl)-1*H*-indole-2-carbonyl)isonicotinic acid: (6.4g, 17.3%), m.p. 272-273 °C (Lit. m.p. 274-275 °C)[1]; IR ν_{max}/cm^{-1} : 2453, 1693, 1671, 1365, 1348, 1272, 1047, 726; δ_H (300 MHz, DMSO-*d*$_6$): 7.28 (1H, s, H-3), 7.38 (1H, overlapping ddd, *J* 8.5, 7.3, 1.2, H-6), 7.55-7.62 (1H, overlapping ddd, *J* 8.5, 7.3, 1.2, H-5), 7.64-7.78 (4H, m, H-7, H-3*, H-4*, H-5*), 7.80 (1H, d, *J* 4.9, H-5′), 8.13-8.23 (3H, m, H-4, H-2*, H-6*), 8.85 (1H, s, H-2′), 8.95 (1H, d, *J* 4.9, H-6′); m/z (ESI$^+$) 407 [(M+H)$^+$, 100%]

4-[(1H-Indol-2-yl)carbonyl]nicotinic acid

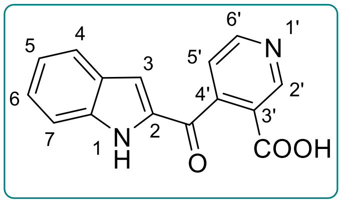

A solution of 4-[{(1-(Phenylsulfonyl)-1*H*-indol-2-yl}carbonyl]-3-pyridinecarboxylic acid (23.3 g, 57.71 mmol) and potassium carbonate (31.7 g, 228.0 mmol) in methanol (400 mL) and water (100 mL) was heated to reflux for 6 hours. Once cooled, the solvent was removed under reduced pressure and water (300 mL) was added. The mixture was cooled to 0 °C and acidified to pH 2 with 37% aqueous hydrochloric acid. The mixture was extracted with ethyl acetate (3 × 300 mL) and the combined organic layers were washed with water (3 × 350 mL) and brine (2 × 350 mL), dried over magnesium sulfate, filtered and concentrated under reduced pressure to give an orange solid (12.5 g, 82.0%). m.p 158-161 °C (Lit. m.p. 159-162 °C)[1]; IR ν_{max}/cm^{-1} : 3384, 3082, 1639, 1522, 1432, 1314, 1267; δ_H (300 MHz, DMSO-*d*$_6$): 6.70 (1H, d, *J* 1.4, H-3), 7.08 (1H, overlapping ddd, *J* 8.2, 7.0, 1.1, H-5), 7.33 (1H, overlapping ddd, *J* 8.2, 7.0, 1.1, H-6), 7.49 (1H, dd, *J* 8.2, 1.1, H-7), 7.61-7.65 (2H, m, H-4, H-5′), 8.92 (1H, d, *J* 4.9, H-6′), 9.15 (1H, s, H-2′), 12.08 (1H, br s, NH); m/z (ESI$^+$) 267 [(M+H)$^+$, 100%]

Indolo[1,2-*b*][2,7]naphthyridine-5,12-dione

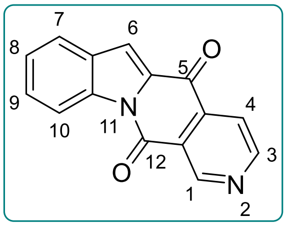

4-[(1*H*-Indol-2-yl)carbonyl]nicotinic acid (12.5g, 47.53 mmol) was heated in acetic anhydride (300 mL) at 85 °C under nitrogen for 20 hours and then at 90 °C for a further 6 hours. The mixture was cooled to room temperature and the solvent was removed under reduced pressure. The residue was cooled to 0 °C and water (300 mL) was added before stirring for 1 hour. The resulting suspension was isolated by vacuum filtration and washed with water (150 mL). The crude product was recrystallised from acetone resulting in a bright orange solid (7.4 g, 63.5%). m.p 195-197 °C (Lit. 196-199 °C)[1]; IR ν_{max}/cm^{-1}: 3043, 1695, 1666, 1545, 1368, 1336, 1243; δ_H (300 MHz, DMSO-*d*$_6$): 7.43 (1H, overlapping ddd, *J* 8.3, 7.2, 1.2, H-9), 7.65 (1H, overlapping ddd, *J* 8.3, 7.2, 1.2, H-8), 7.81 (1H, s, H-6), 7.88 (1H, d, *J* 8.3, H-10), 8.00 (1H, d, *J* 5.0, H-4), 8.49 (1H, d, *J* 8.3, H-7), 9.12 (1H, d, *J* 5.0, H-3), 9.46 (1H, s, H-1); m/z (ESI$^+$) 249 [(M+H)$^+$, 100%]

5,11-Dimethyl-6*H*-pyrido[4,3*b*]carbazole (Ellipticine)

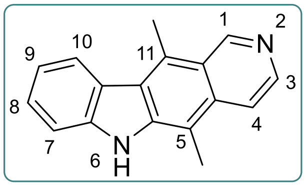

To a solution of Indolo[1,2-*b*][2,7]naphthyridine-5,12-dione (3.06 g, 12.01 mmol) in THF (360 mL) maintained at -105 °C was added MeLi (13.25 mL, 1.8 M, 24.0 mmol) dropwise. The reaction mixture was kept at -105 °C for a further 1 hour and then allowed to warm to room temperature overnight. The reaction was quenched with water (10 mL) and the solvent was removed under reduced pressure. The residue was dissolved in absolute ethanol (360 mL) and treated with sodium borohydride (9.00 g, 240 mmol) and set to reflux under nitrogen for 6 hours. A second addition of sodium borohydride (9.00 g, 240 mmol) was added, and the reaction was allowed continue under reflux for a further 42 hours. Once cooled, the solvent was removed under reduced pressure and the crude product was purified by column chromatography, eluting in dichloromethane-methanol (100:0-97:3) yielding a yellow solid (1.89 g, 64.1 %). m.p >300 °C (Lit. 311-315 °C)[1]; IR ν_{max}/cm^{-1}: 3145, 3074, 2975, 1614, 1597, 1464, 1319, 1306, 1241; δ_H (300 MHz, DMSO-d_6): 2.80 (3H, s, CH_3-5), 3.27 (3H, s, CH_3-11) 7.27 (1H, overlapping ddd, *J* 8.1, 6.8, 1.1, H-9), 7.54 (1H, overlapping ddd, *J* 8.1, 6.8, 1.1, H-8), 7.56 (1H, d, *J* 8.1, H-7), 7.93 (1H, dd, *J* 6.0, 0.7, H-4), 8.39 (1H, d, *J* 8.1, H-10), 8.44 (1H,d, *J* 6.0, H-3), 9.71 (1H, s, H-1), 11.38 (1H, s, NH); m/z (ESI$^+$) 247 [(M+H)$^+$, 100%]

1. G.W. Gribble, M.G. Saulnier, J.A. Obaza-Nutaitis, D.M. Ketcha, A versatile and efficient construction of the 6H-pyrido[4,3-b]carbazole ring system. Syntheses of the antitumor alkaloids ellipticine, 9-methoxyellipticine, and olivacine, and their analogs, The Journal of Organic Chemistry 57(22) (1992) 5891-5899.

^{1}H NMR Data of **26**

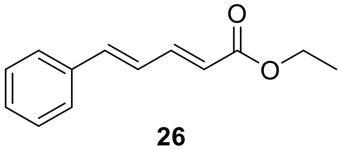

26

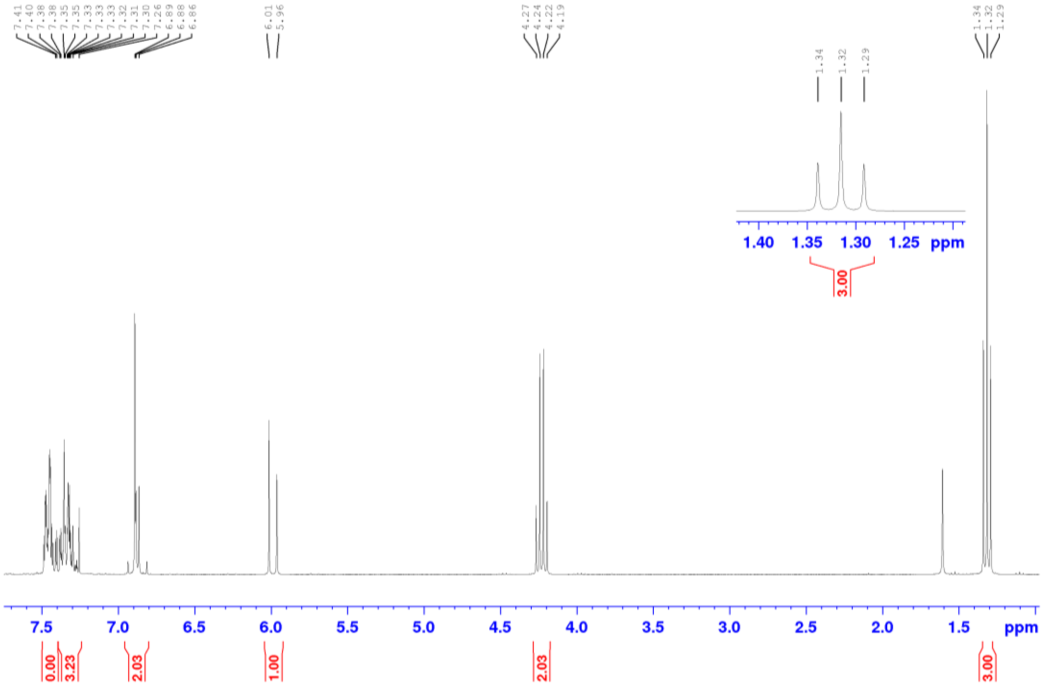

^{1}H NMR Data of **27**

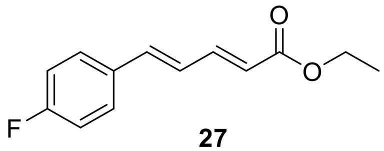

27

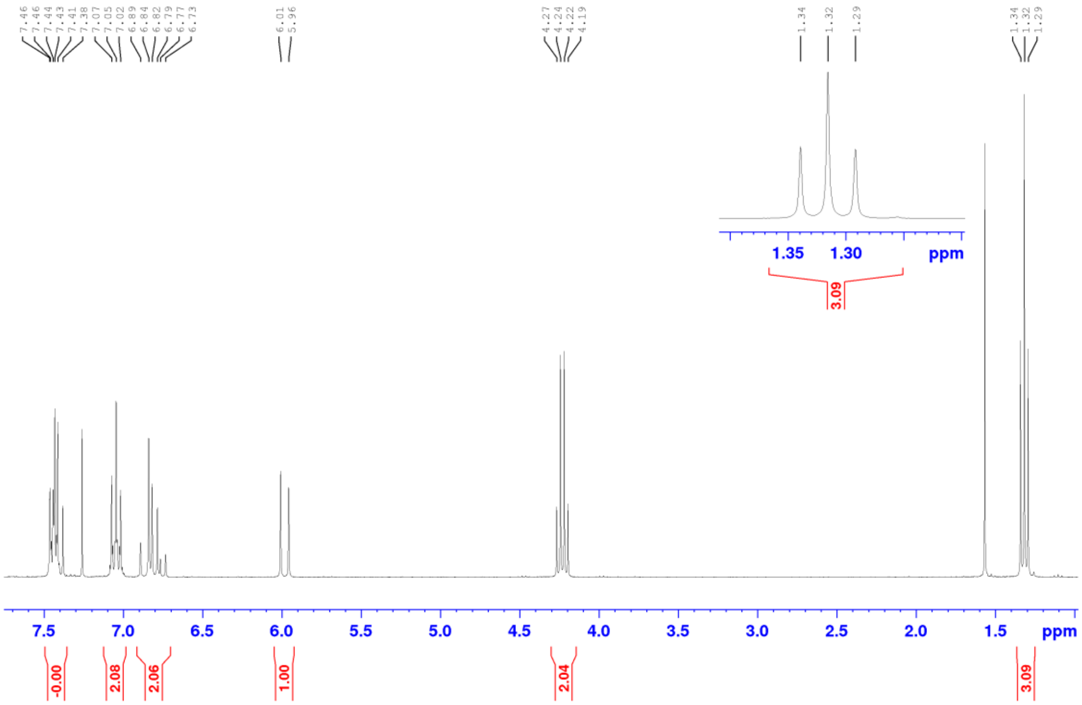

^{1}H NMR Data of **28**

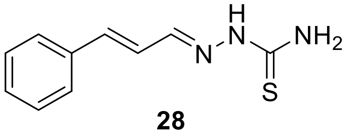

28

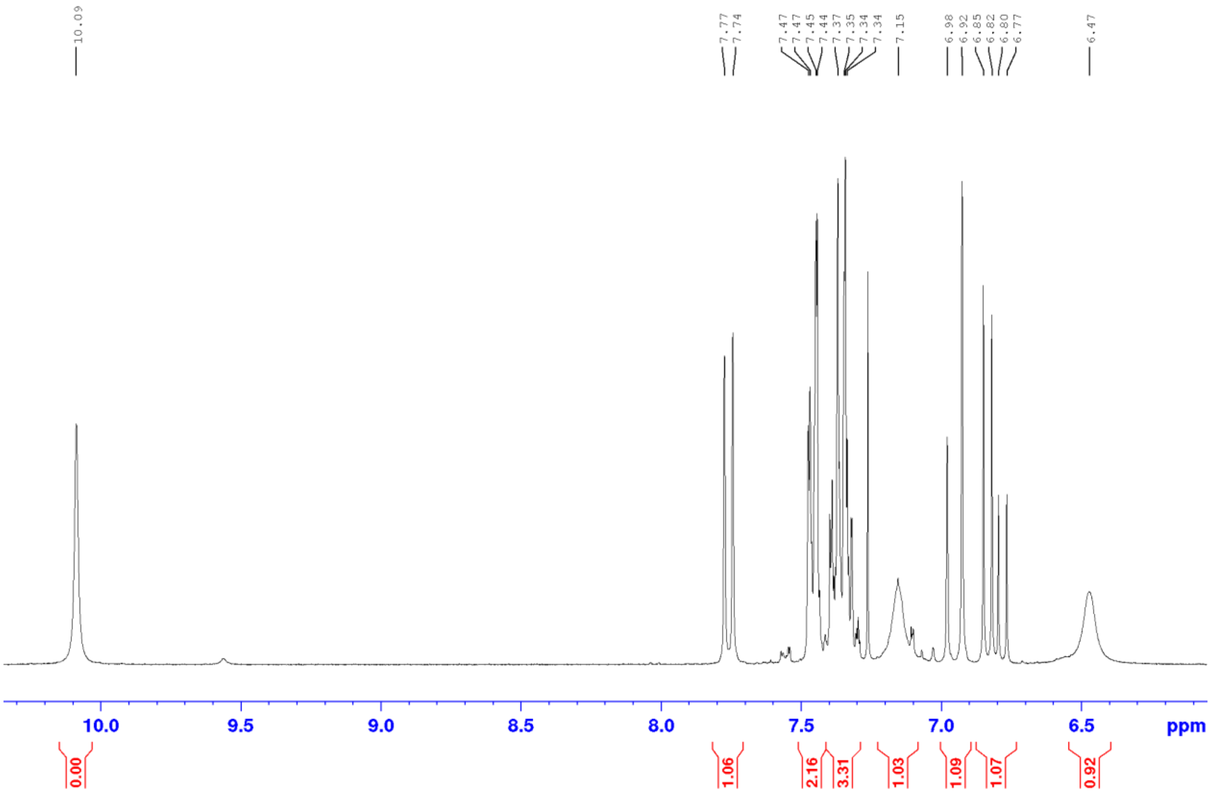

^{1}H NMR Data of **29**

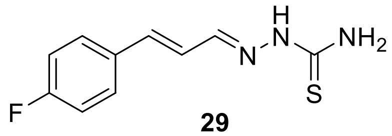

29

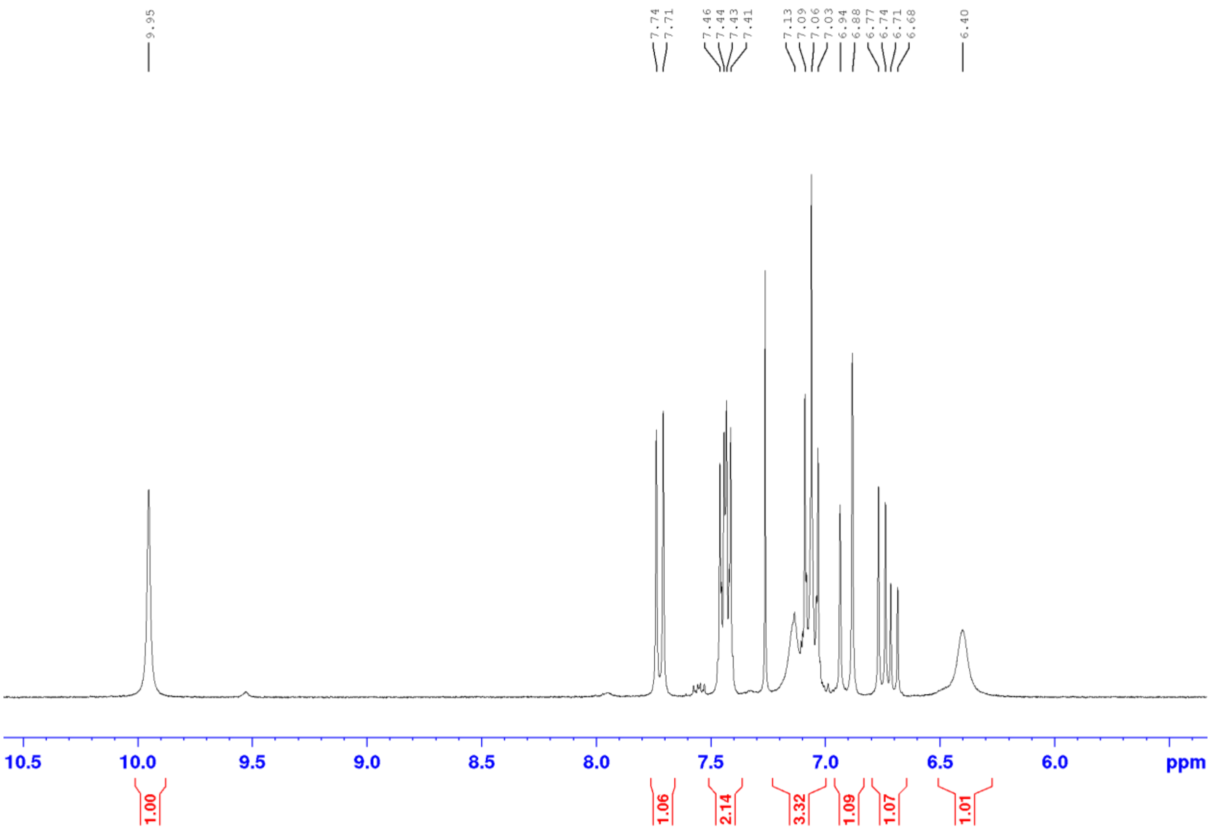

^{13}C NMR Data of **29**

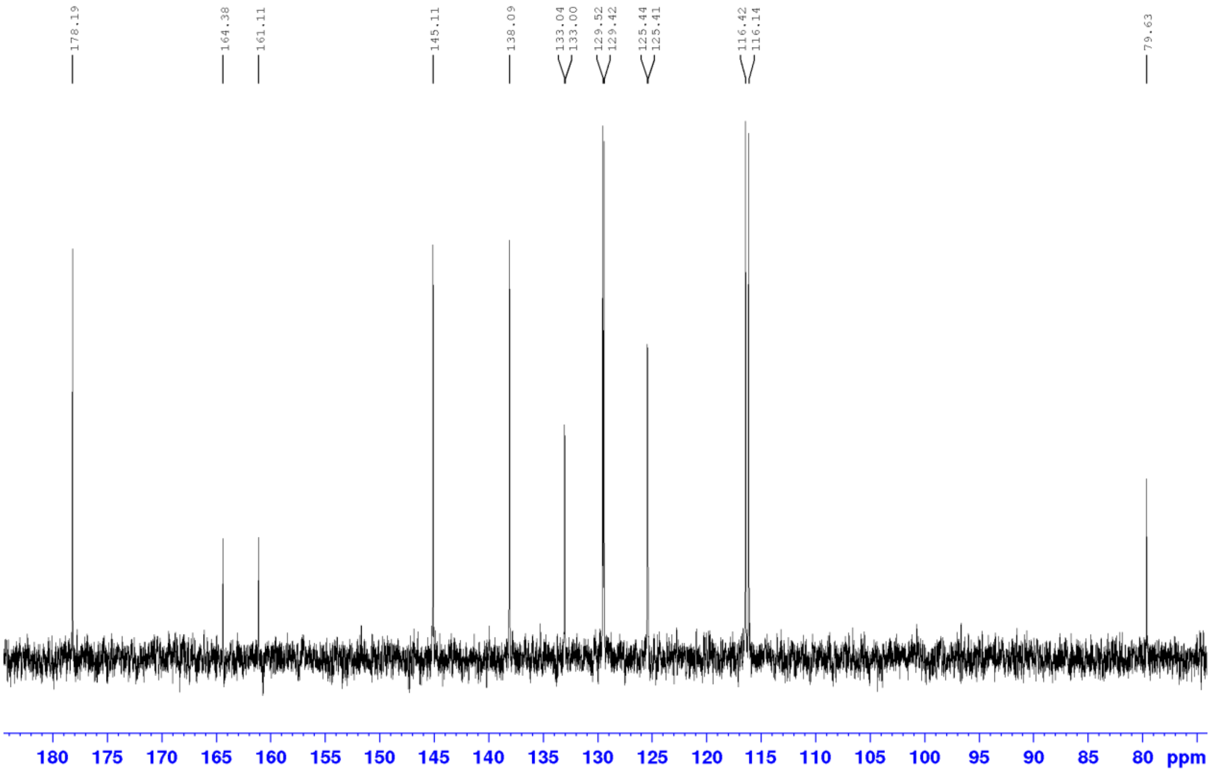

^{1}H NMR Data of **30**

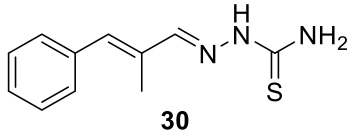

30

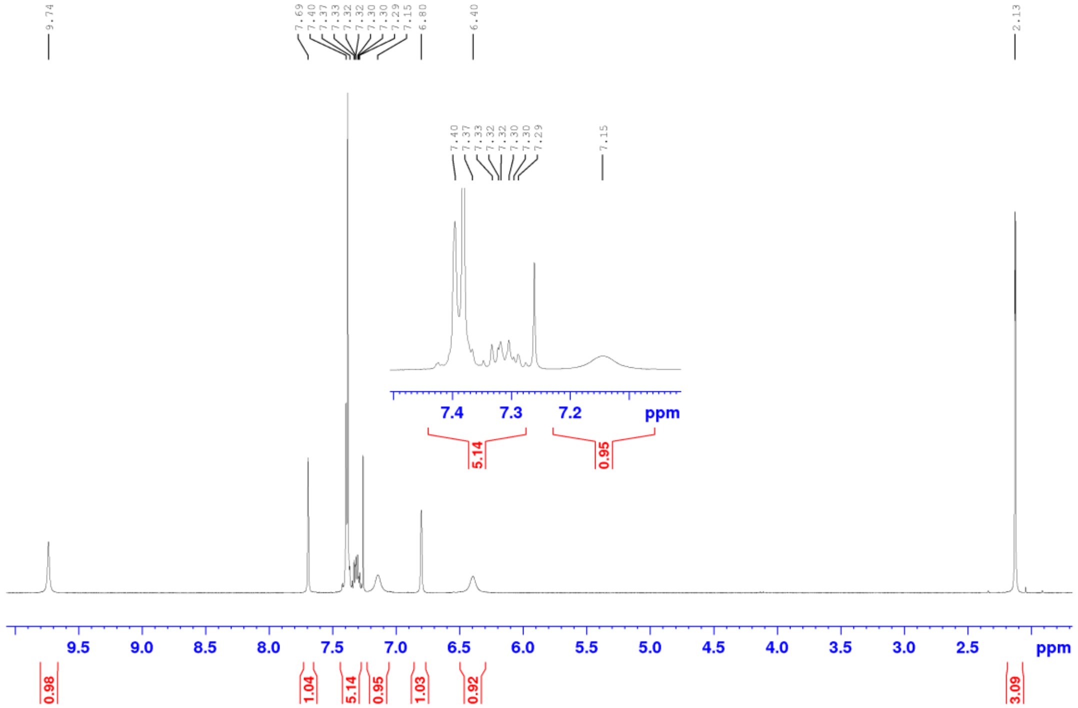

^{1}H NMR Data of **31**

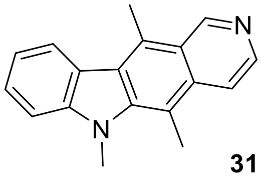

31

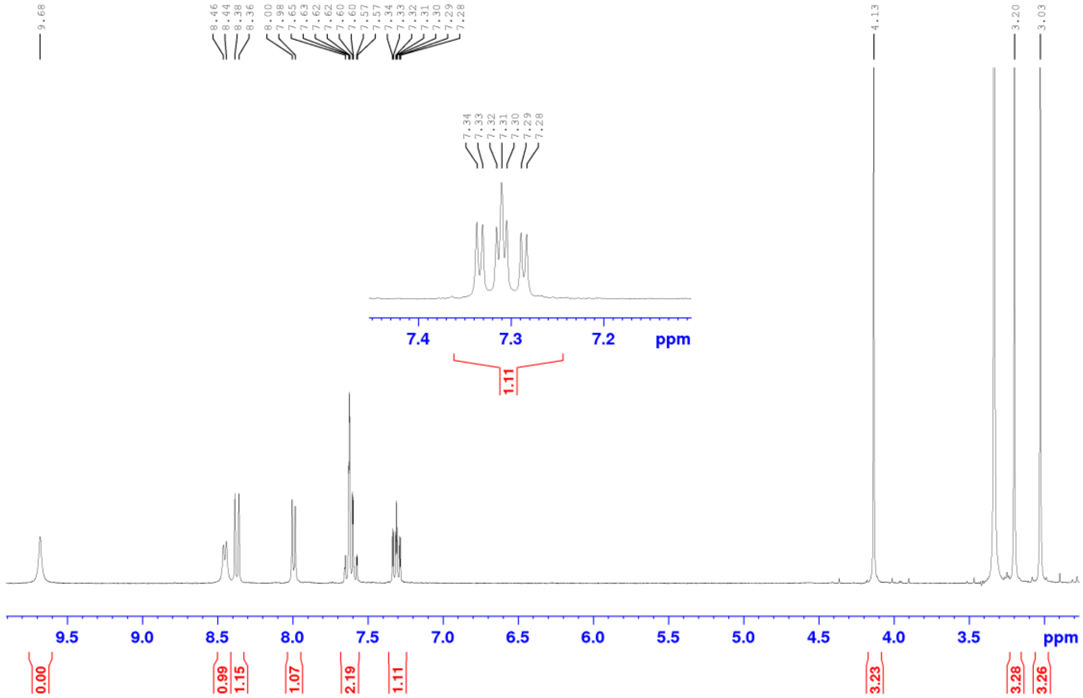

^{1}H NMR Data of **32**

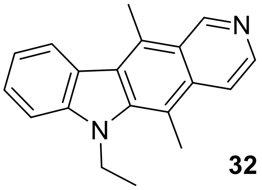

32

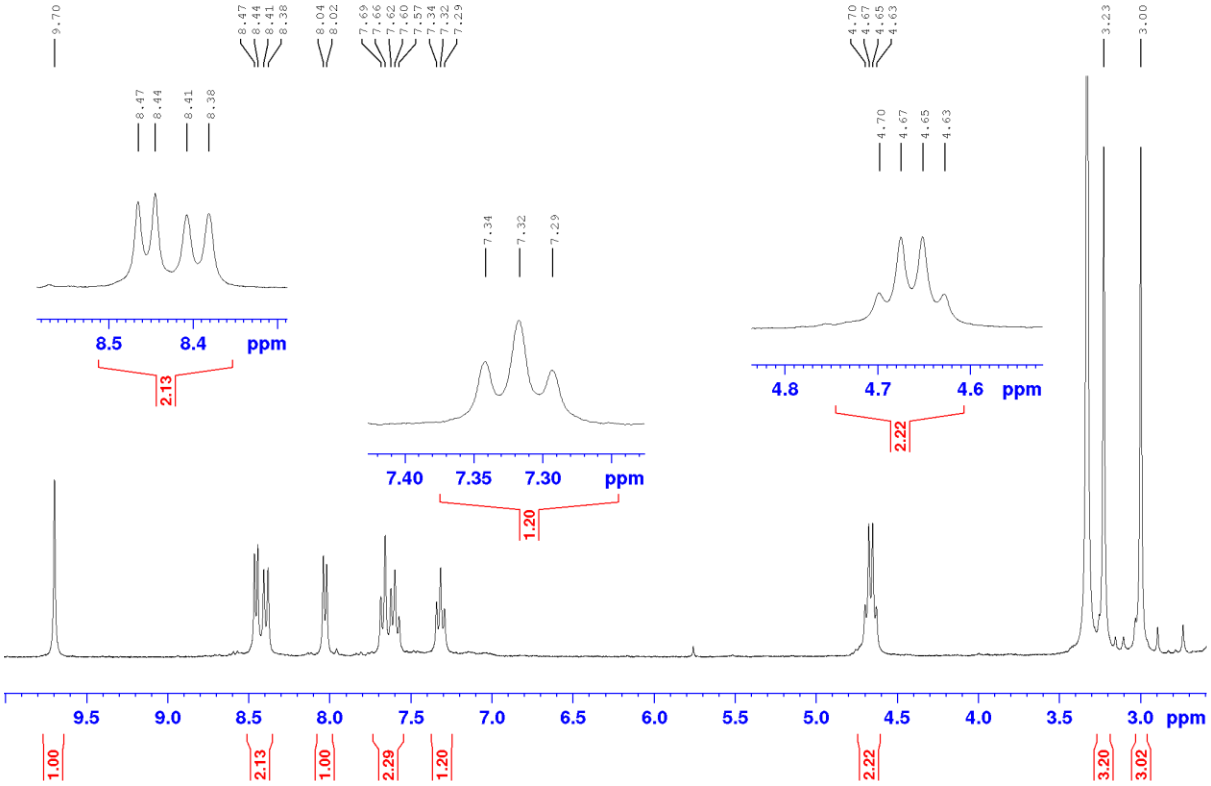

^{1}H NMR Data of **33**

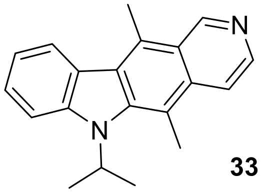

33

^{1}H NMR Data of **33**

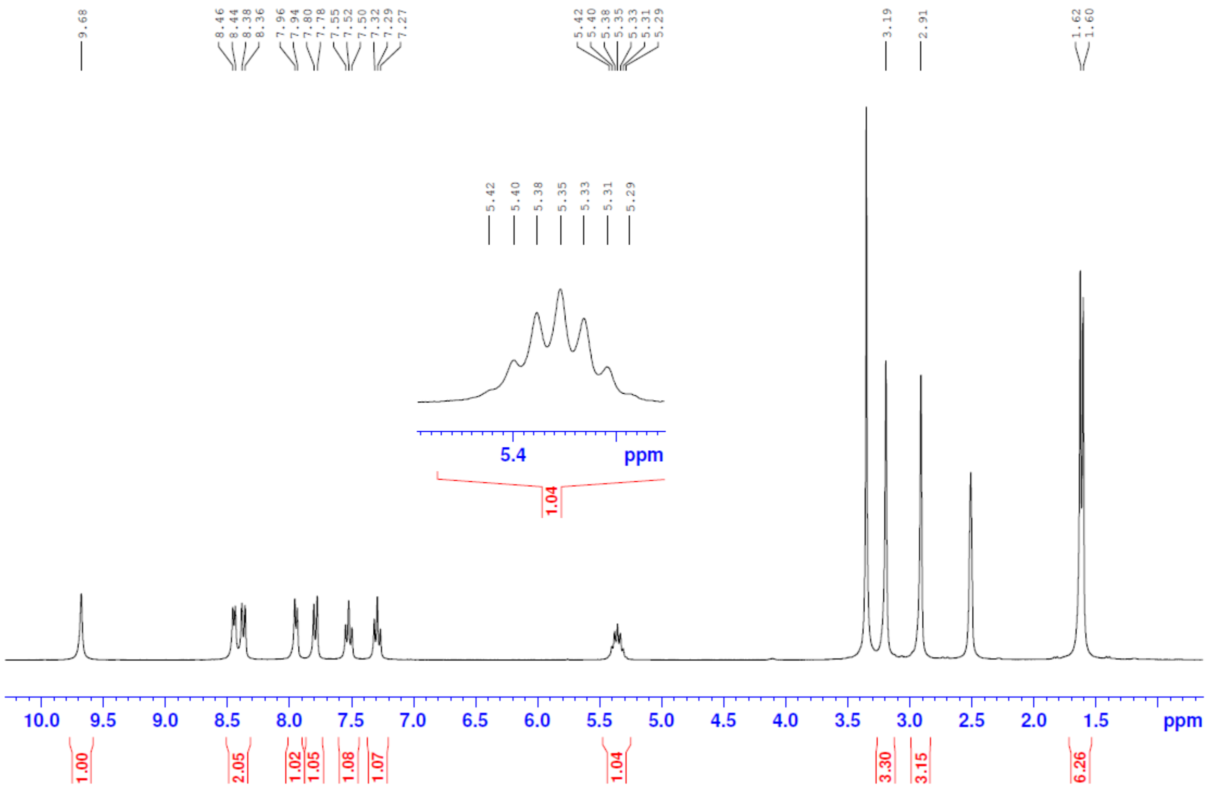

^{1}H NMR Data of **35**

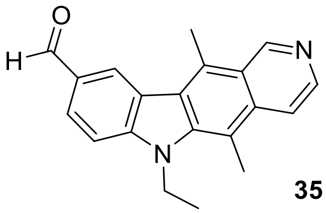

35

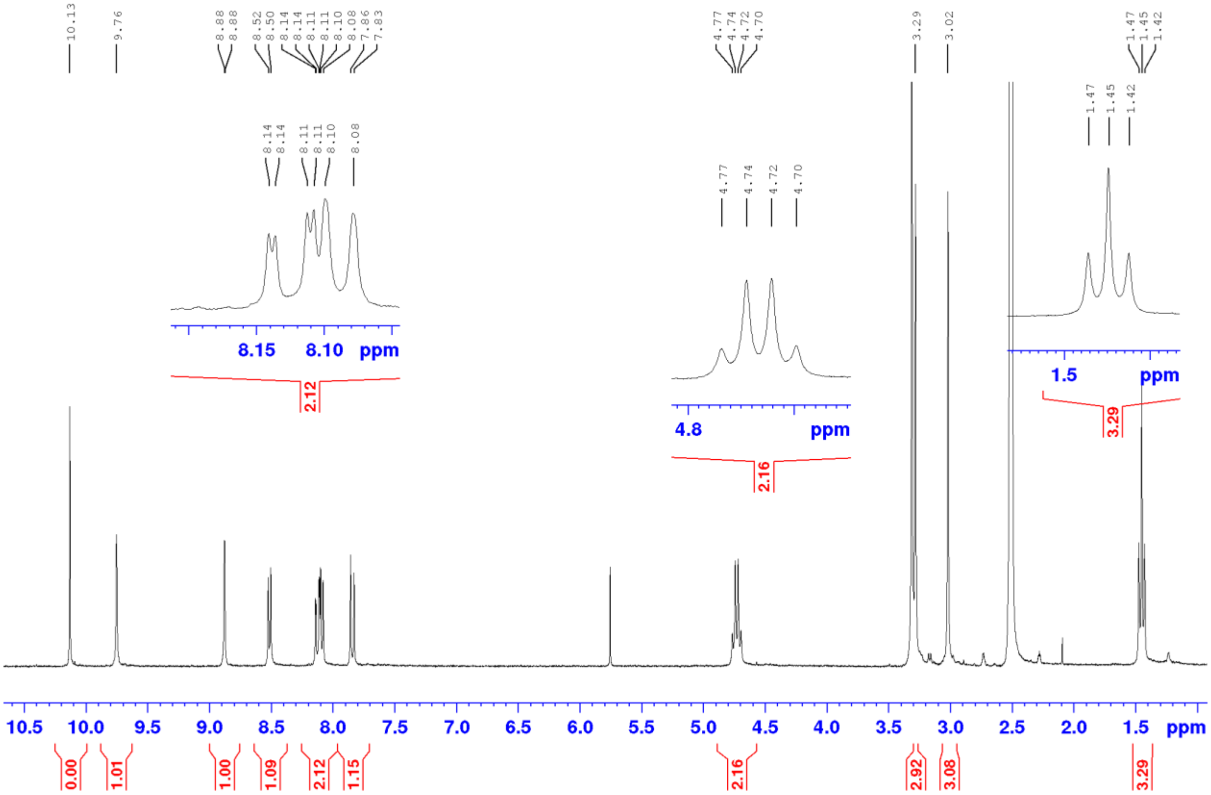

^{1}H NMR Data of **36**

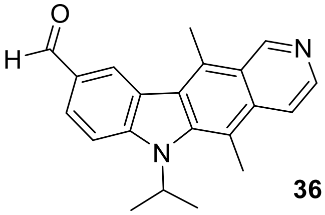

36

^{1}H NMR Data of **36**

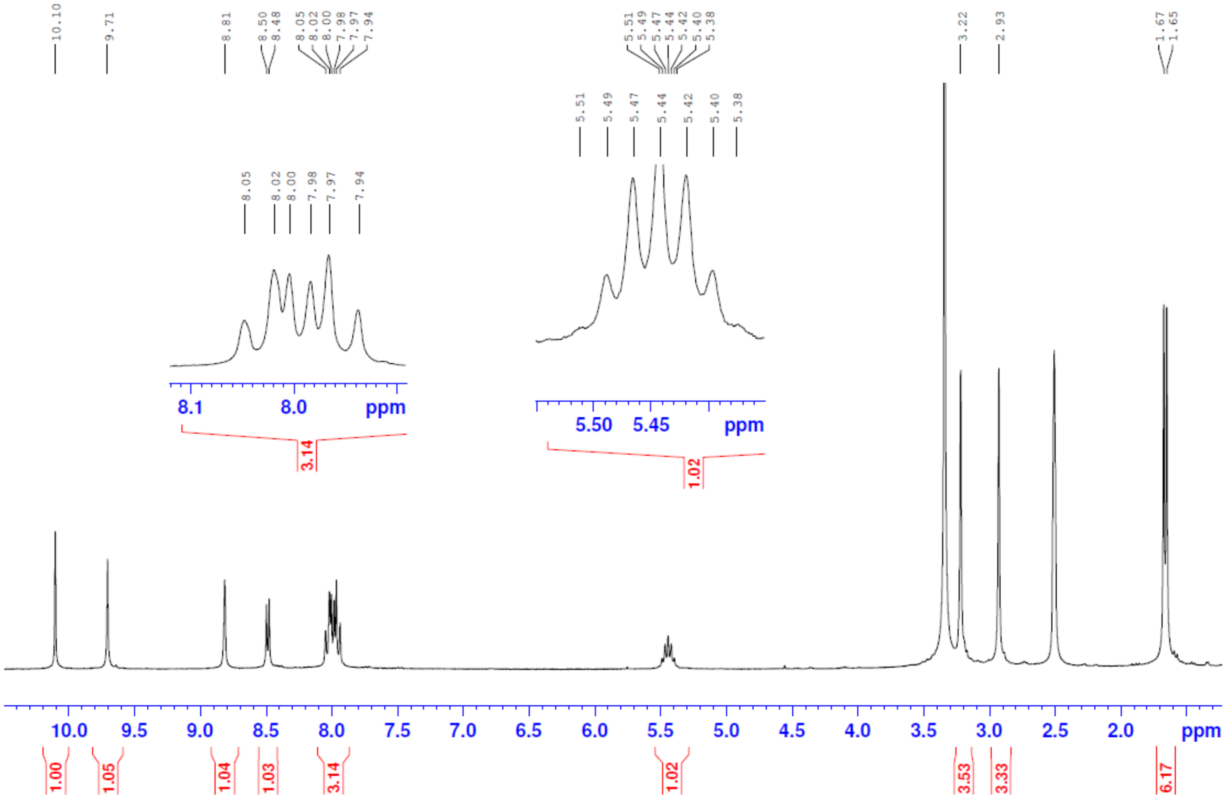

*Mycelial Growth Inhibition of cinnamaldehyde (**7**), hydrocinnamaldehyde **6** and Mancozeb **1***

Cinnamaldehyde (**CA**) Rye B agar *P. Infestans* 88069: Day 5

CA 2mM

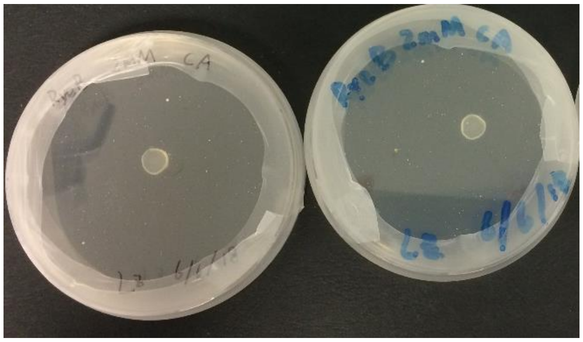

CA 1mM

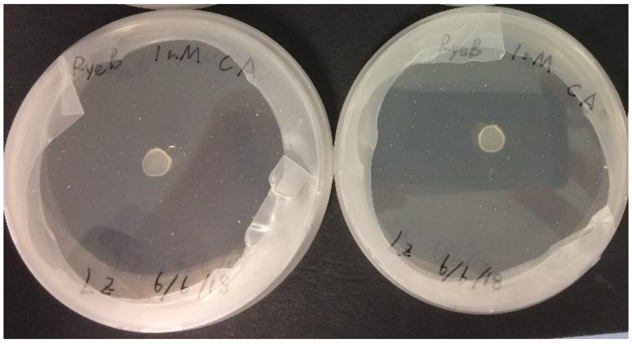

CA 0.5mM

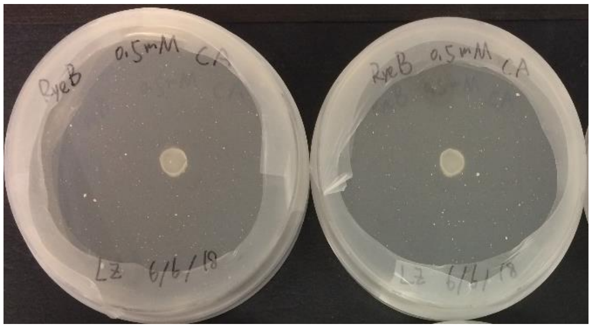

CA 0.25mM

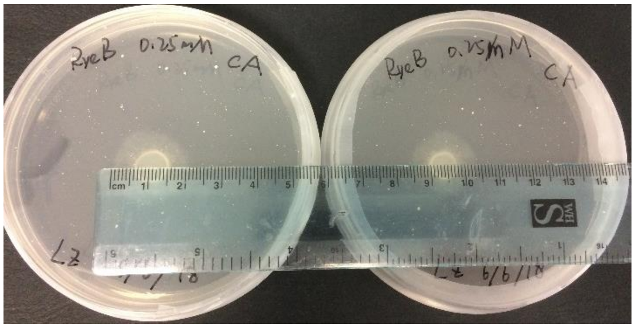

CA 0.1mM

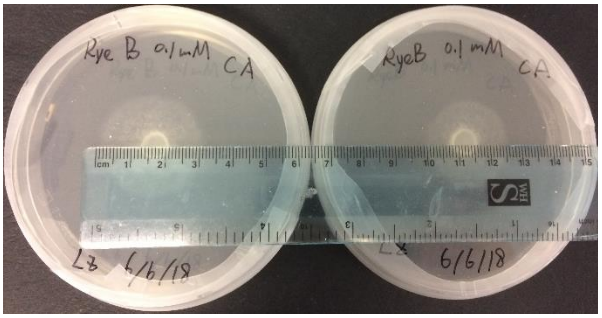

EtOH 0.01%

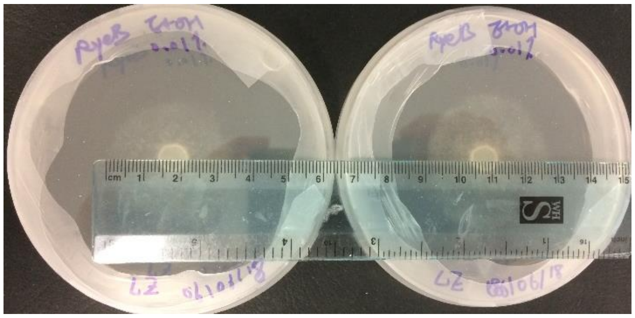

Hydrocinnamaldehyde (**HCA**) Rye B agar *P. Infestans* 88069: Day 5

HCA 2mM

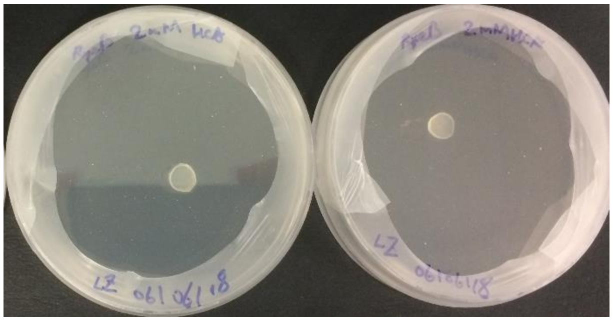

HCA 1mM

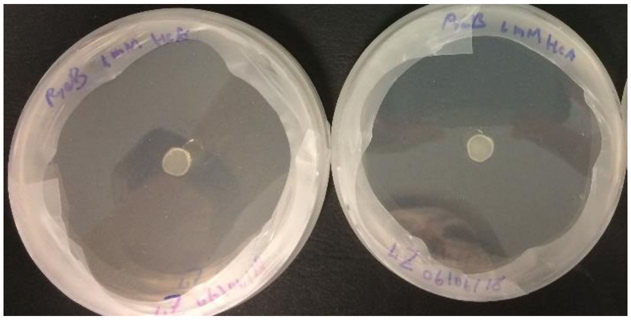

HCA 0.5mM

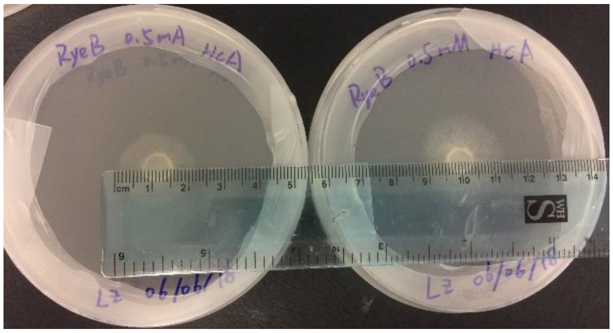

HCA 0.25mM

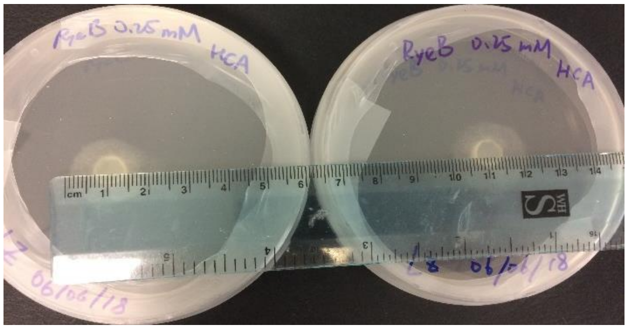

HCA 0.1mM

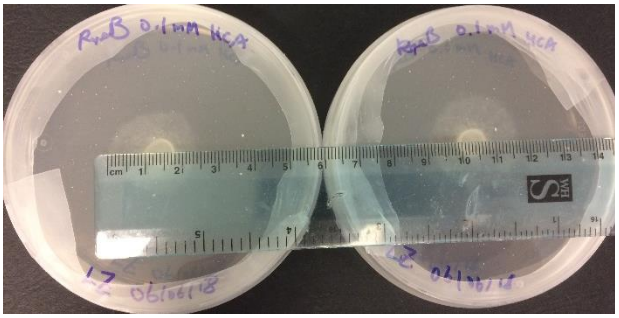

EtOH 0.01%

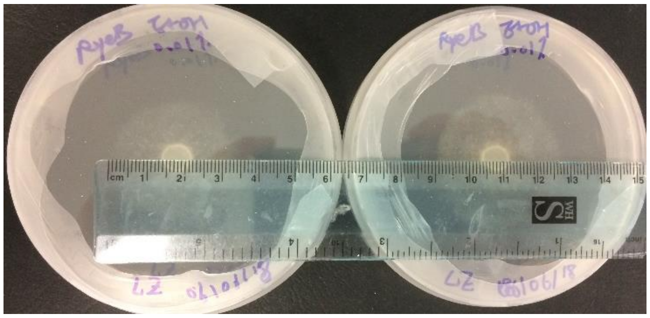

Mancozeb (MB) Rye B agar *P. Infestans* 88069: Day 5

MB 200uM

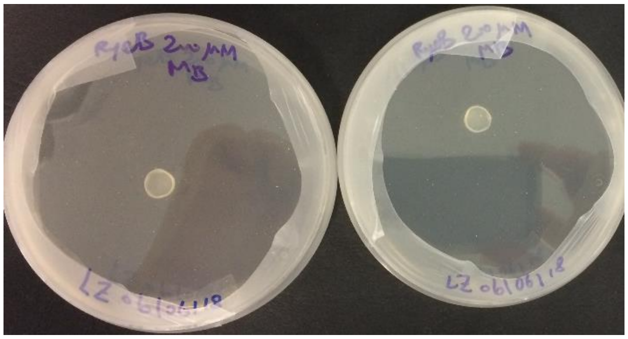

MB 100uM

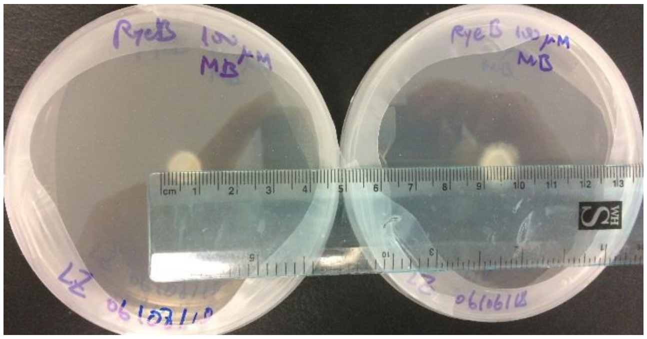

MB 50uM

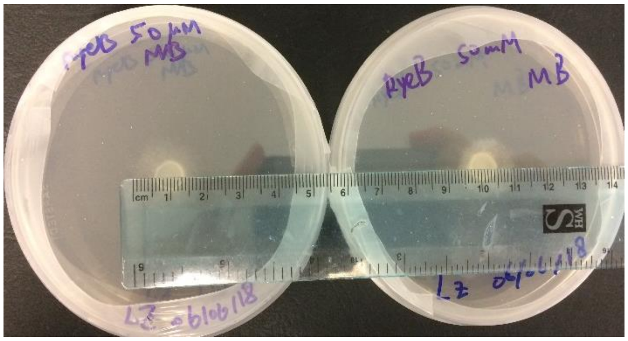

MB 25uM

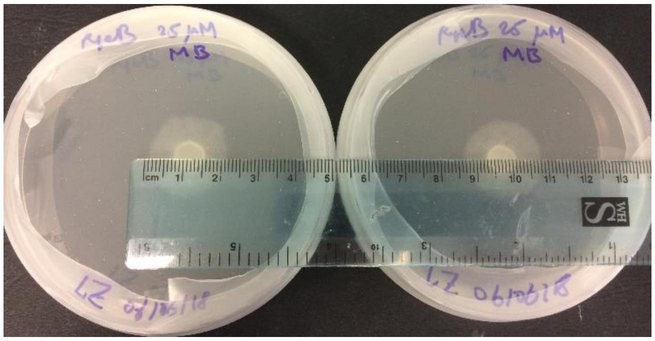

MB 10uM

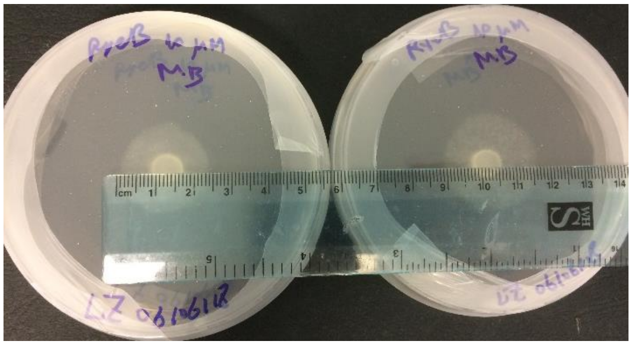

DMSO 0.05%

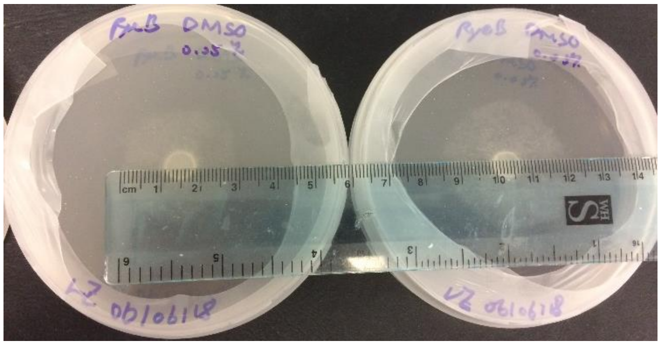

Supplement: Supplementary file 1 [file pathogens-09-00542-s001.pdf]
